# What matters when exploring fidelity when using health IT to reduce disparities?

**DOI:** 10.1186/s12911-021-01476-z

**Published:** 2021-04-07

**Authors:** Margaret A. Handley, Jerad Landeros, Cindie Wu, Adriana Najmabadi, Daniela Vargas, Priyanka Athavale

**Affiliations:** 1 Department of Epidemiology and Biostatistics, University of California, San Francisco, San Francisco, USA; 2 Division of General Internal Medicine, Department of Medicine, Center for Vulnerable Populations, University of California, San Francisco, San Francisco, USA; 3 University of California, Berkeley School of Public Health, Berkeley, USA

**Keywords:** Program evaluation, Language-concordant care, Health equity, Health IT, Health coaching

## Abstract

**Background:**

Implementation of evidence-based interventions often involves strategies to engage diverse populations while also attempting to maintain external validity. When using health IT tools to deliver patient-centered health messages, systems-level requirements are often at odds with ‘on-the ground’ tailoring approaches for patient-centered care or ensuring equity among linguistically diverse populations.

**Methods:**

We conducted a fidelity and acceptability-focused evaluation of the STAR MAMA Program, a 5-month bilingual (English and Spanish) intervention for reducing diabetes risk factors among 181 post-partum women with recent gestational diabetes. The study’s purpose was to explore fidelity to pre-determined ‘core’ (e.g. systems integration) and ‘modifiable’ equity components (e.g. health coaching responsiveness, and variation by language) using an adapted implementation fidelity framework. Participant-level surveys, systems-level databases of message delivery, call completion, and coaching notes were included.

**Results:**

96.6% of participants are Latina and 80.9% were born outside the US. Among those receiving the STAR MAMA intervention; 55 received the calls in Spanish (61%) and 35 English (39%). 90% (n = 81) completed ≥ one week. Initially, systems errors were common, and increased triggers for health coach call-backs. Although Spanish speakers had more triggers over the intervention period, the difference was not statistically significant. Of the calls triggering a health coach follow-up, attempts were made for 85.4% (n = 152) of the English call triggers and for 80.0% (n = 279) of the Spanish call triggers (NS). Of attempted calls, health coaching calls were complete for 55.6% (n = 85) of English-language call triggers and for 56.6% of Spanish-language call triggers (NS). Some differences in acceptability were noted by language, with Spanish-speakers reporting higher satisfaction with prevention content (*p* = < 0.01) and English-speakers reporting health coaches were less considerate of their time (*p* = 0.03).

**Conclusions:**

By exploring fidelity by language-specific factors, we identified important differences in some but not all equity indicators, with early systems errors quicky remedied and high overall engagement and acceptability. Practice implications include: (1) establishing criteria for languge-equity in interventions, (2) planning for systems level errors so as to reduce their impact between language groups and over time; and (3) examining the impact of engagement with language-concordant interventions on outcomes, including acceptability.

*Trial Registration* National Clinical Trials registration number: CT02240420 Registered September 15, 2014. ClinicalTrials.gov.

## Background

To improve evidence-based practice, practice-based interventions must balance adaptations to local circumstances with attempts to maintain external validity. It is implied but not always explicitly described that for an intervention or evidence-based practice (EBI) to be considered evidence-based, findings need to be replicated with fidelity, even while adaptations occur [1]. According to Carroll et al., fidelity refers to “the degree to which an intervention is delivered as intended” and is used to determine to what extent an intervention has been adequately ‘replicated’[2]. Process monitoring and understanding fidelity are critical for planning. Determining factors associated with implementation success and failure are cornerstones of implementation science [3].

Fidelity most often involves attention to content, dose and duration, which can be thought of as general measures of protocol adherence [4, 5]. Increasingly, *context* is considered in regards to intervention fidelity [6], resulting in increased adaptations to local conditions. As a result, *moderating* factors affecting fidelity, such as context, participant responsiveness, intervention complexity are included in fidelity models [6–8]. The increased focus on adaptations and context means that a more detailed exploration of intervention fidelity is possible [7, 8]. Recent theoretical work in this area [8], has proposed reviewing both fidelity and adaptation in the context of a ‘value equation’ which focuses more on the final desired outcomes beyond intervention effects, linking three concepts: (1) the end product should emphasize overall value rather than only the intervention effects, (2) implementation strategies are a means to create ‘fit’ between EBIs and context, and (3) transparency is required. Additionally, to ensure that health equity is improved and not worsened from the delivery of health IT interventions to vulnerable populations, such as those with limited English proficiency and poor access to healthcare resources, attention to equity in this value equation context is important [6, 9, 10],

Language-concordant care, delivered through patient counselling or health coaching, is a critical predictor of improved self-management outcomes [11–13] and can address disparities in health outcomes [14, 15]. Technology-assisted diabetes self-management and prevention programs, including those that provide patient-centered supports, have expanded significantly in the last decade with a myriad of approaches including: web-based programs [16, 17], SMART phone applications or apps [18, 19], telephone-based automated call programs, (often referred to as Automated Telephonic Self-Management Support, or ‘ATSM’ or Interactive Voice Response) which blend narratives content with queries that require patients to respond via touch tone with the information going to a central location for review [20–24]. However, studies have noted that language-concordant intervention delivery is limited [25] highlighting a gap in using Health IT to reduce disparities in ways to ensure the digital divide is not exacerbated for low-income, limited English proficient populations.

It is in this context, the expansion of multi-lingual health IT delivery for diabetes prevention support [26, 27], that we developed and implemented the STAR MAMA intervention (Support via Telephone Advice/Resources Sistema Telefonico de Apoyo y Recoursos) [28–30], the first program of its kind to delivery a bilingual ATSM-based program to post-partum women with a history of gestational diabetes (GDM), a significant predictor of subsequent on-set of type 2 *diabetes mellitis*. In particular, we explore implementation outcomes and moderating factors for the STAR MAMA program and build on the value outcome concept described above in relationship to fidelity using an equity lens. The following areas are the focus of this equity-based evaluation: (1) moderators of implementation fidelity for STAR MAMA such as variability by language and over time; (2) participant responsiveness, (3) acceptability, and (4) quality of delivery, including health coaching.

## Methods

### Study summary

**Fig. 1:**
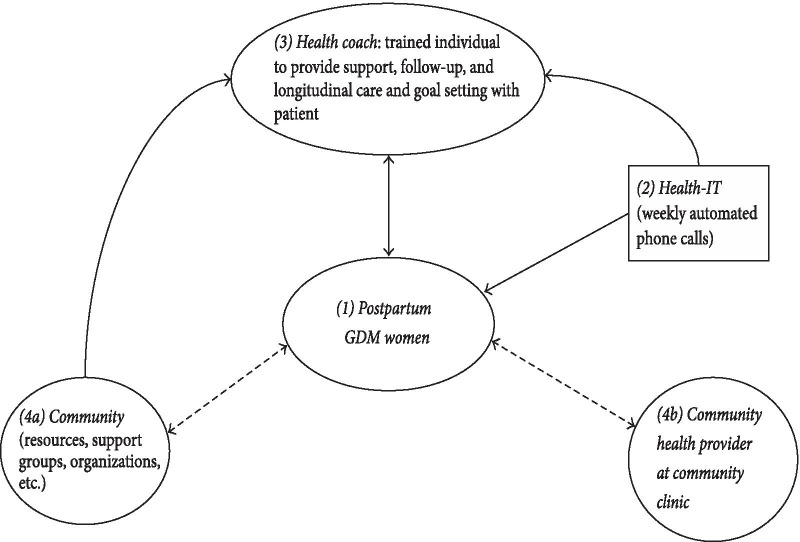
STAR MAMA Program. (1) The woman is enrolled in the program 6 weeks post-partum. (2). The system “pushes” weekly calls using touch tone responses, which a health coach reviews (3) in a weekly report and then engages the patient in follow-up health coaching telephone calls, based on pre-determined ‘triggers’ for weekly responses. (4) Health coaches provide linkages to clinic and community resources

STAR MAMA is a 20 week bilingual (English and Spanish) ATSM-based program which combines automated 3–5 min weekly calls including queries and narratives with ‘live’ follow-up calls from a language-concordant health coach (plus opt-in text messages) to encourage diabetes prevention behaviors among post-partum women with recent gestational diabetes (Fig. 1). We apply an established implementation framework for fidelity evaluation [6, 7] and a range of data sources in order to explore the impact on 9–12 month outcomes, using a type-1 hybrid implementation effectiveness study and a randomized clinical trial design [31]. Women recruited from safety net sites were individually randomized during a baseline visit at the end of their pregnancies to either STAR MAMA calls or to an education only arm. Health outcomes were evaluated using structured interviews and medical records review, and included: weight loss (BMI reduction), breast-feeding duration and the percentage of women actively engaged in chronic disease risk reduction behaviors (such as increased physical activity and decreased consumption of sugar sweetened beverages and program acceptability for those in the intervention arm. The trial enrolled 181 post-partum women receiving health care in safety net settings in the San Francisco Bay Area, between 2014 and 2018. Study sites included Zuckerberg San Francisco General Hospital (ZSFGH), SF-Women Infant Child Programs, and Sonoma County- Women Infant Child Programs and a Federally Qualified Health Center. All study procedures were approved by the University of California, San Francisco Committee on Human Research. Participants were given gift cards valued at $135 total as reimbursement for their participation in baseline and follow-up interviews.

STAR MAMA was developed using a theory-informed approach, applying the Capability Opportunity and Motivation (COM-B) model and related Behavior Change Wheel [32], as well as Social Cognitive Theory alongside a stakeholder engagement process to improve the relevance and reach of the intervention content for the linguistically diverse populations receiving it [28, 29]. Based on stakeholder input, STAR MAMA incorporated the Diabetes Prevention Program (DPP) [33] combining content focusing on health at the individual level (participant and infant), and socio-ecological drivers affecting health behavior, such as food insecurity and social support/social isolation (Appendix). The content includes a mix of narrative storytelling showcasing supportive messaging about challenges (e.g. stress, mood, fussy babies), questions that ask about behaviors for the health coaches to review responses to (e.g. “*Are you having trouble breastfeeding?, press 1 if yes and 0 if no*”), and tips, in the form of recipes, text links to videos and community resources. Topics focused on behaviors related to diabetes prevention (weight loss, healthy eating, physical activity, glucose screening, breast feeding, stress and mental health) and on key areas of infant health in the first 6 months (vaccination timing, breastfeeding, fussiness, sleep, introduction of food). The intervention was delivered weekly beginning at 6 weeks post-partum at a day and time selected by the participant, and lasting 20 weeks, after which a follow-up interview was completed over the phone or in-person.

The structure of STAR MAMA includes both a “push” of diabetes prevention messages directed at improving adherence to diabetes prevention related behaviors to women, and a “pull” of engaging participants with health coaching call backs, based on participant responses to behavioral questions (e.g. “*how many sugar sweetened drinks did you have in the last 7 days? enter the number of drinks*”) and pre-determined trigger thresholds for health coaching call backs (e.g. reporting more than 1 day drinking sugar sweetened beverages, or ‘yes’ to difficulty with breastfeeding). Primary health outcomes from the study will be reported elsewhere.

### Fidelity analysis—overview of fidelity-related outcomes

**Fig. 2:**
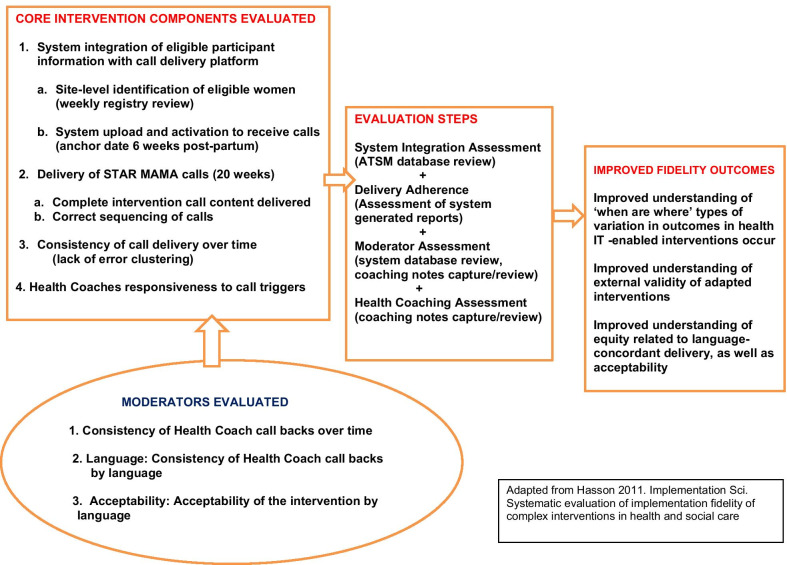
Conceptual framework for fidelity evaluation: STAR MAMA

The goals of the fidelity analysis are to determine to what extent the STAR MAMA program was delivered as intended, for core intervention components related to: (1) System Integration: *completeness and correct timing* of the STAR MAMA delivery system such that women first received their calls as intended beginning 6 weeks post-partum, at their preferred day and time; (2) Intervention Delivery: *correct sequencing* of the weekly calls, the “push” of the intervention; (3) Call Consistency: for weekly calls over the intervention period; and (4) Health Coach Responsiveness: for attempted call backs for call triggers generated by the STAR MAMA system. (Table 1). All measures were evaluated for variation by language as a potential equity moderator of fidelity (Fig. 2). Acceptability was also included in the fidelity assessment as a moderator—the rationale being that participant engagement in the intervention could affect the health coaches responses. For example, it would be important to understand which program aspects had higher and lower acceptability and identify where there might be variation by language.

**Table 1:** Core STAR MAMA intervention components and moderating factors evaluated

Domain	What is required for high fidelity	Fidelity assessment questions	Specific outcome	[Data Source]
*Core fidelity component assessment*				
1. System Integration (combining participant registry with intervention delivery platform)	(a) Complete registration and uploading of GDM patient details to ATSM system (after baseline enrolment/randomization) (b) Activation of the ATSM system to initiate intervention delivery (timed to 6 weeks post-partum)	(a) Was the participant-level data integrated into the ATSM system prior to 6 weeks post-partum? (b) Was the STAR MAMA start correctly implemented for the first call to be delivered 6 weeks post-partum?	(a) % of enrolled women in trial at each clinical site, with data uploaded to the ATSM delivery platform (b) % of intervention starts correctly linked to delivery date	[System-generated weekly report]
2. Intervention delivery	Correct ATSM ‘push’ of weekly intervention content to each participant, at a pre-specified time and day, based on participant preferences	Did the ATSM system correctly delivery weekly call content to participants? (a) All the calls were sent as planned (b) Delivery of all intervention weeks (completeness) (c) Delivery of correct sequencing of intervention (alignment with intervention logic for post-partum period/infant development)	(a) % of calls correctly delivered (b) % of participants delivered/not delivered all 20 weeks of calls (c) % of participants with delivered weeks in the correct sequence for all 20 weeks	[System-generated weekly and daily reports]
3. Consistency of intervention delivery over time	Consistency of ATSM ‘push’ of weekly intervention content across the study intervention period	Did the ATSM system result in error clustering? Or were errors spread out over time across weeks and over the study period?	% of participants with an early (weeks 1–5) vs later (≥ week 6) missed week delivery	[System-generated weekly and daily reports]
4. Health coach responsiveness to participant ‘triggers’	Responsiveness of health coaches to participant triggers generated by touch tone responses to calls	Did health coaches call back participants whose daily reports indicated a trigger warranting further follow up?	% of women with at least one documented trigger who subsequently received a health coach initiated call attempt	[System-generated weekly and daily reports] and [Health Coach Database]
*Moderating factors*				
Health coach consistency of outreach over time	Level of fluctuation in call-back attempts over time by health coaches	Did health coaches make call-back attempts consistently over the study intervention period?	% of triggers over time that received an attempted call back	[System-generated weekly and daily reports] and [Health Coach Database]
Language equity: health coach consistency of outreach for participant language and enrolment site	Level of fluctuation in call-back attempts by language or site	Did health coaches make call-back attempts similarly for Spanish and English speaking participants and for enrolment sites?	% of triggers by language, over time, that received an attempted call back	[System-generated weekly and daily reports] and [Health Coach Database]
Language equity: acceptability of the STAR MAMA calls and health coaching package	N/A	How acceptable was the intervention to enrolled women? Would they do it again or refer others?	Levels of acceptability reported at follow-up after the program was over	[Interview data]

Integration of the participant enrollment registry with the STAR MAMA delivery system was estimated as the percent of enrolled women who were subsequently uploaded to the Health IT intervention delivery platform prior to the target start date of the women’s calls, beginning 6 weeks post-partum. This time-sensitive activity involved site-level identification of the eligible women with GDM (with confirmation at 32 weeks gestation) through review of weekly clinic trackers and databases, contacting women post-partum to determine their preferences for call dates and times, uploading preferences, and activating the STAR MAMA call initiation.

System delivery of the STAR MAMA call content was measured by counting the number of calls with the correct content delivered (vs. “incorrect”), in the correct sequence (vs. “skipped”), and in the correct language for the patient based on weekly system generated reports. We also measured the consistency of call delivery over time, evaluating whether all 20 weeks of STAR MAMA calls were delivered, and whether any errors appeared over the 20 weeks (such as missed weeks), and variation by language.

### Fidelity analysis—moderators

We explored potential moderators of the quality of delivery including: health coach consistency of attempted call backs over time and health coach consistency regarding attempted call backs by participant language and enrollment site (i.e.consistency over time and program week, minutes on each trigger type, and call back rates by language). For participant responsiveness, the proportion of the target group that engaged with the intervention, and variation by language were examined. We calculated the counts of calls picked up, calls completed, and whether the health coach attempted the protocol-driven follow-up call. Acceptability measures were derived from 9 to 12 month semi-structured follow-up interviews, and included: overall acceptability with the STAR MAMA program; acceptability of different aspects of the pushed content, acceptability of health coaches; level of involvement of other close friends/family in program activities; and indication of intention to do another program like STAR MAMA again.

### Fidelity analysis—data analysis

Data sources for the fidelity analysis include: (1) an automatic system report (call attempts, week of the message, call duration, triggers indicated); (2) project-driven information (health coach notes, database of daily reports); and (3) interview data. Engagement measures for the fidelity analysis included: delivery of ATSM calls, ATSM call responses by participants, and health coachingsummary notes, including topics discussed, resources referred, action plans made and length of call. 

## Results

**Fig. 3:**
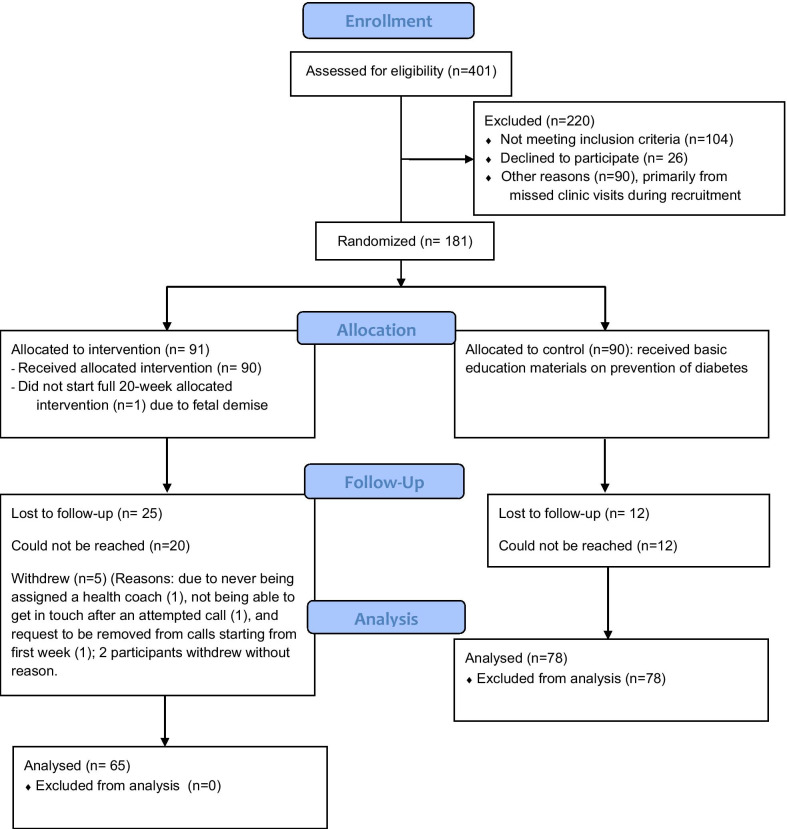
STAR MAMA CONSORT flow diagram

Of the 181 women who were recruited, 90 were randomized to the STAR MAMA ATSM calls and 91 to the education only arm (see CONSORT diagram Fig. 3). Participant mean age was 31.5 years, 96.6% of participants are Latina and 80.9% were born outside the US. Among those receiving the ATSM calls 55 received the calls in Spanish (61%) and 35 English (39%). Of those in the ATSM arm, 81 women (90%) completed one or more of the 20 weeks of the program. Five participants withdrew (3 English-speaking and 2 Spanish-speaking, total of 6% of enrolled population) and 4 were lost to follow-up. Sixty-one women in the intervention arm who completed follow-up interviews are included in the acceptability assessment.

### Overall STAR MAMA call completion: program adherence

**Fig. 4:**
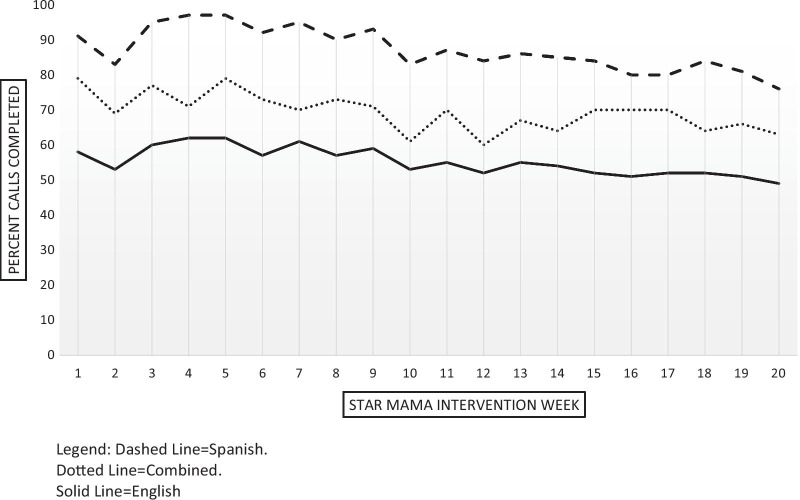
STAR MAMA completed calls by week and language

Overall STAR MAMA engagement was moderately high, with a mean of 11.8 weeks (standard deviation (SD) = 7.0) completed out of 20 total weeks in the program (Fig. 4). 81 of the 90 women randomized to calls completed at least the first week of the program (90%). Fifty-four (66.7%) women completed at least half (10 +) of the weeks in the program and forty (50%) women completed at least 70% (14 weeks) of the program. Spanish speaking participants had higher levels of call completion than did English-speaking participants (among those completing 10 or more calls), but the result was not statistically significant. The mean number of calls completed was 12.4 (SD 6.6) for Spanish speakers and 10.7 (SD 7.5) for English speakers.

### Delivery of the STAR MAMA program: system integration

There were no errors in the system integration components evaluated with all participants correctly uploaded to the platform, and for the activation of calls to begin at 6 weeks after the confirmed delivery date.

### Delivery of the STAR MAMA program: intervention delivery completeness

We separate out program call completion assessments into two categories: system-driven and participant-driven. Of the 81 participants who completed some or all of the 20 weeks, there were a total of 1620 calls programmed to be pushed by the ATSM system. At the system-level, there were 31 (1.9%) missed calls, 5 (0.3%) incorrect calls, 29 (1.7%) skipped calls, and 73 (4.5%) error messages. The number of unique patients affected was 21 (25.9%) for missed calls, 5 (6.2%) for incorrect calls, 23 (28.4%) for skipped calls, and 30 (37%) for error messages. There were 666 (41.1%) partially completed calls, in which the participant did not complete the entirety of the call response prompts but did some of them. There were 9 (11.1%) participants who did not complete the call in at least one of the weeks.

### Delivery of the STAR MAMA program: intervention delivery sequencing

A sequencing issue (“skipped” week) occurs when the wrong week’s content was delivered in a certain week. There were a total of 29 calls sent that contained the incorrect week of content. This affected 23 unique patients (28% of participants who completed the STAR MAMA calls); the majority (n = 20) experiencing one skipped week.

### Delivery of the STAR MAMA program: consistency over time

Participants who started when the program was first implemented experienced more call issues, as the program was working out the technical issues. For example, the first 17 participants experienced 56% of all the problems with the STAR MAMA call delivery and sequencing, and the first 25 participants experienced 70% of all the call issues. The patients who experienced the most call issues experienced 3–4 missed calls over the course of their 20-week program; these patients were within the first 17 participants to start the program. Looking at total errors, a significant proportion were experienced in the first 5 weeks (41.1% of all errors) of the 20 weeks total program duration. These system-level errors for delivery completeness, sequencing and consistency over time did not disproportionately affect either language group (data not shown).

### Health coaching triggers and participant call-backs

The mean number of weeks where a health coaching call back was triggered was 6.5 (SD = 4.1, median = 7), representing about one-third of the calls that could have triggered a health coaching call-backs (n = 18 weeks). The mean number of weeks with a completed health coaching call-backs was 3.2 (SD = 3.01, median = 2). The total duration of the health coaching calls for each patient varied considerably, with a mean of 41.8 min (SD = 38.9, median = 29). By week five of the program, 43% of all triggers had occurred. Seventy-four percent of all triggers indicated occurred in the first 11 weeks of the program.

**Fig. 5:**
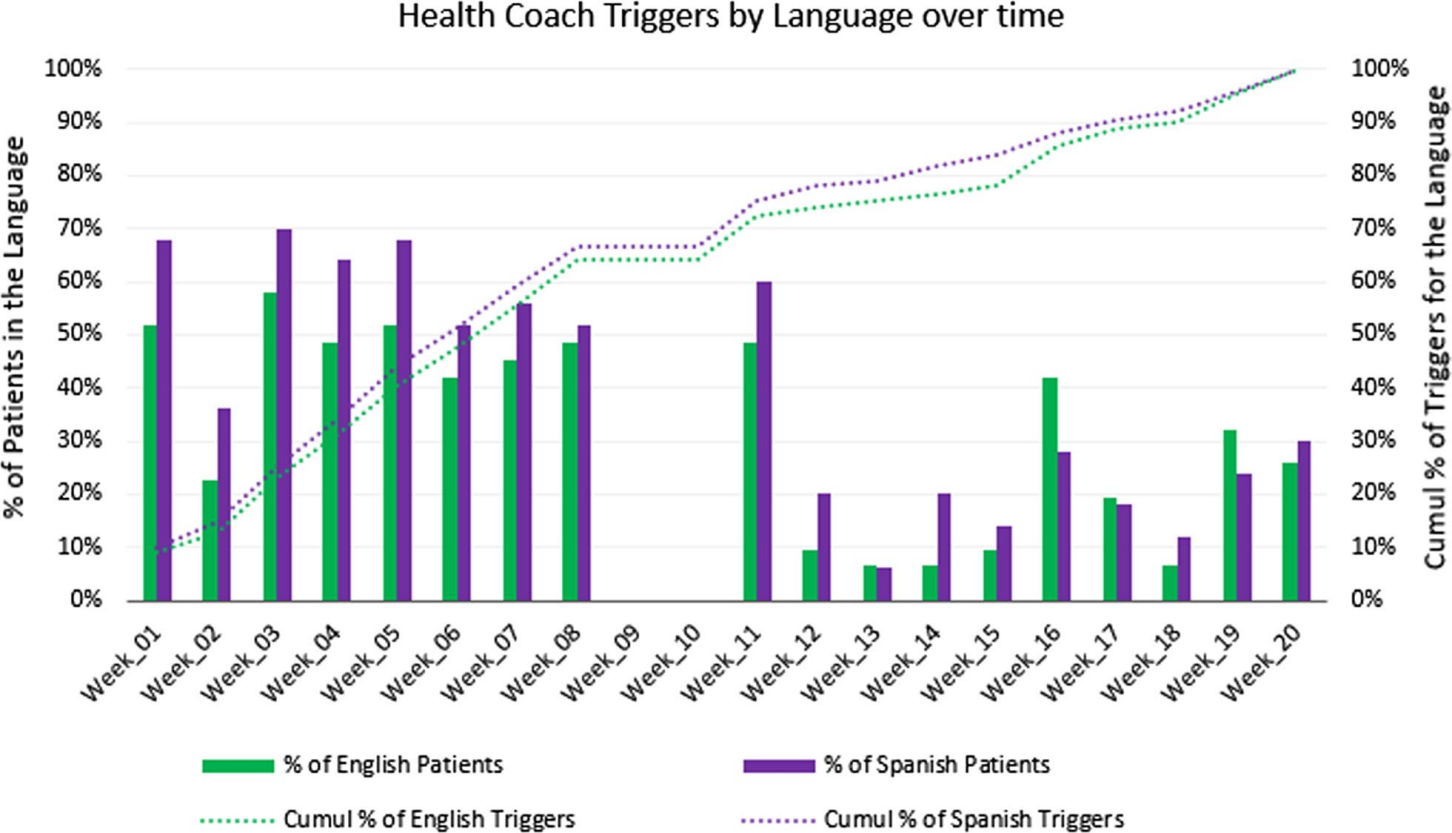
Health coach triggers for STAR MAMA: by language and over time

**Fig. 6:**
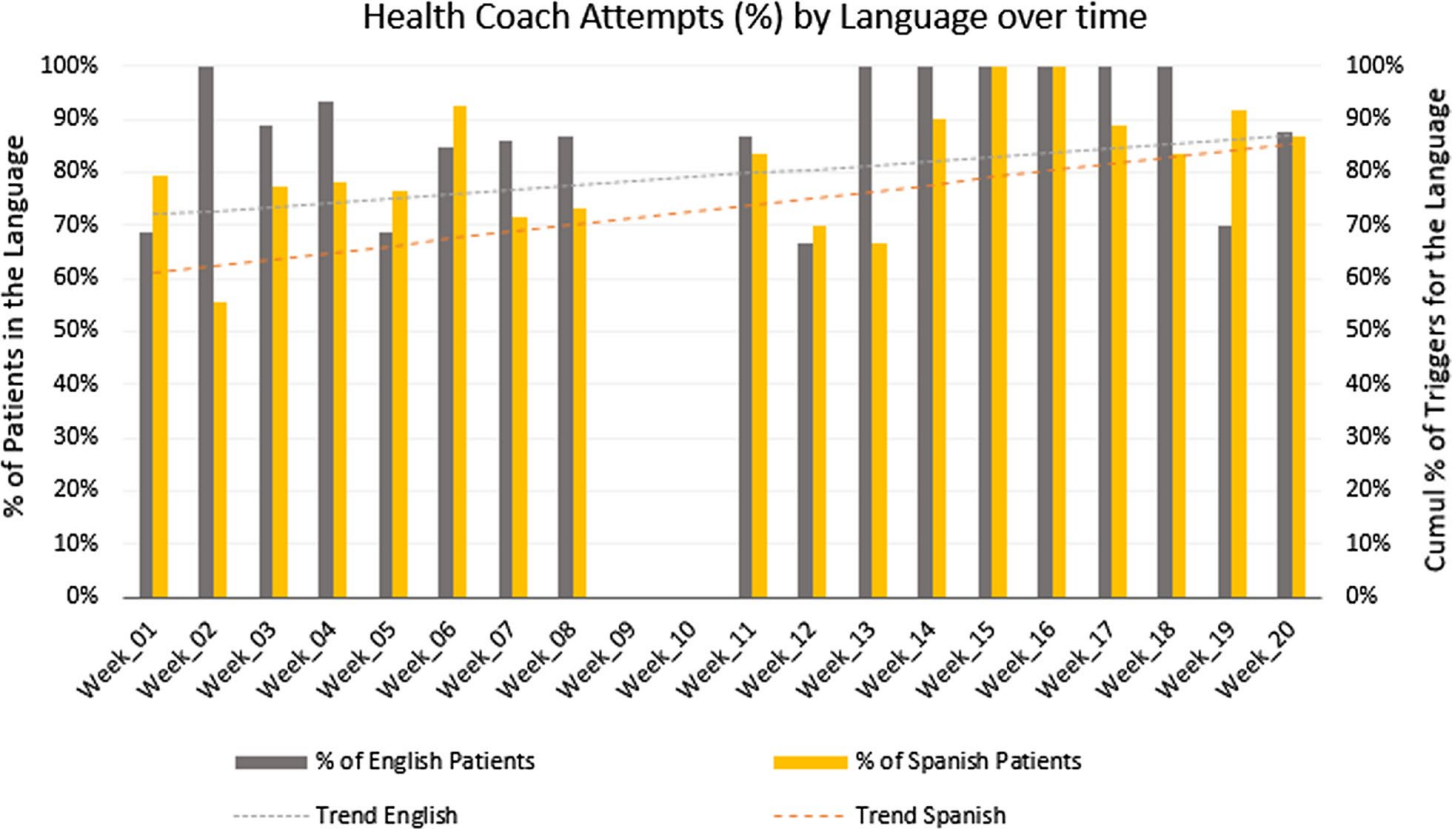
Health coach attempts for STAR MAMA over time

Of all 1,620 possible calls, English speakers (n = 31, 38% of participants) should have received 620 calls, and Spanish speakers (n = 50, 62% of participants) should have received 1,000 calls (see Fig. 5). Of the calls in English, 29% (n = 178) triggered a health coach follow-up, while of the calls in Spanish, 35% (n = 349) triggered a health coach follow-up. There were many more triggers in the first several weeks of the STAR MAMA intervention than later on in the program, especially among Spanish-speakers. Although Spanish speakers had more triggers than did English-speakers, the difference was not statistically significant. Of the calls that triggered a health coach follow-up, a call-back attempt was made for 85.4% (n = 152) of the English call triggers and for 80.0% (n = 279) of the Spanish call triggers. Of those with attempted calls, health coaching calls were complete for 55.6% (n = 85) of English-language call triggers and for 56.6% of Spanish-language call triggers. Again, there were no differences by language in attempted or completed health coaching call-backs. Additionally, attempted call backs were consistent over time and by language of call trigger (Fig. 6).

### Acceptability

Overall acceptability was high for STAR MAMA calls (Table 2) and in general did not differ by language, with a few notable exceptions including: agreement that the program provided “useful information on diabetes prevention and baby care” (Spanish speakers reporting higher agreement, *p* < 0.01); and English speakers were less likely to report the health coaches were considerate of their time (*p* = 0.03). Ninety percent of the 61 women interviewed in the call arm reported they would do the program again, with no difference by language. Over half (55.6%) had shared the program ideas with friends and 75.8% had engaged a partner in some of the STAR MAMA content. Two-thirds reported that the number of weeks was ‘fine’, with a third indicating the program was “too long”.

**Table 2:** Acceptability indicators among STAR MAMA call participants completing follow-up (n = 61)

	Combined (N = 61)		English-Speaking participants		Spanish speaking participants		*p* value
	% agree program worked fine/no call problems	% agree there were 1 or more call problems	% agree program worked fine/no call problems	% agree there were 1 or more call problems	% agree program worked fine/no call problems	% agree there were 1 or more call problems	
*Technical indicators-acceptability*							
Quality of sequencing of calls	82.8	17.2	90.5	9.5	78.4	21.6	0.30
Call length	88.5	11.5	91.5	8.5	81.8	18.2	0.06
Audio quality	91.6	8.3	90.9	9.1	92.1	7.9	1.00
Clarity of call	98.3	1.7	100	0	97.4	2.6	1.00
Text ‘opt in’ quality*	67.2	32.8	66.7	33.3	67.6	32.4	1.00

	% Agree	% Disagree, or neither agree or disagree					
*Call content indicators-acceptability*							
Information useful for diabetes prevention	86.0	14.0	63.5	36.5	97.0	3.0	**0.003**
Information useful for baby care	91.8	8.2	73.3	26.7	100	0	**0.006**
Information useful for losing weight	88.4	11.6	94.1	5.9	85.7	14.2	0.65
Information useful for increasing physical activity	94.2	5.8	94.1	5.9	94.2	5.7	1.00
Information useful for eating healthy/nutrition	98.0	2.0	100	0	97.2	2.8	1.00
Information useful for reducing intake of sugars	98.0	2.0	94.1	5.9	100	0	0.32
*Call content indicators-social support*							
Supported my feelings as a new mom	93.3	6.7	86.4	13.6	97.4	2.6	0.14
Gave me ideas to find other people in my life to support me in diabetes prevention	76.7	23.2	63.6	36.3	84.2	15.8	0.11
*Health coaching acceptability indicators*							
Health coaches helped me for diabetes prevention	78.4	21.6	75	25	80	20	0.72
Health coaches helped me for baby care	92.3	7.7	94.1	5.9	91.4	8.5	1.00
Health coaches helped me feel supported as a new mom	92.3	7.7	88.3	11.7	94.3	5.7	0.58
Health Coaches were considerate of my time	96.1	3.9	83.3	16.7	100	0	**0.03**

*n = 41 women opting in to text messages

## Discussion

In this paper we report on core fidelity metrics for the STAR MAMA study and explore the relationship of language and other moderators to fidelity of both systems level implementation and ‘on the ground’ live health coaching responses. We found that while there were many early systems level errors that resulted in missed weeks of delivery, these were not affected by language and decreased to minimal over time. We also found that a higher proporortion of Spanish-speaking women engaged with the program and completed a greater mean number of weeks, but these differences were not statistically significant. This higher engagement by non-English speakers is consistent with other work we have done in safety net settings for diabetes care [21] as is the higher level of satisfaction with content reported in Table 2 (such as ‘useful information for baby care’ and ‘useful information for diabetes prevention’). In this fidelity analysis, we found relatively high levels of health coach triggers, with about 30% of the calls triggering, with approximately half of these having a completed call-back. We did not find significant differences in these coaching activities by language, and high overall levels of satisfaction with the program.

Although there were few effects of a language differential in the evaluation, there were some trends in differences by language in systems-level problems as well as in health coaching interactions. We believe that it is critical to determine to what extent efforts to increase diverse populations in health IT interventions are well adapted to the local context and to this end, it is important to evaluate the errors inherent in any automated processes designed to reach a wider range of participants, and their potential lasting impact across the intervention period. The findings in this study extend the work on fidelity assessments both by exploring language as a moderator across all implementation components as well as by framing language-concordance as an equity component for consideration.

### Study limitations

There are several limitations to this study. Information on high and low adopters, by language would have provided critical insights into necessary modifications. We did find high acceptability across language groups but drivers of dissatisfaction, are less specified in the quantitative descriptive analysis. Additionally, conducting modeling to explore the relationship between fidelity and health outcomes was out of scope for this study, since it was a pilot, with a relatively small intervention arm sample size. Also, it is possible that there are complex relationships between moderators, as suggested by Carroll 2007 [2] which were not examined. Similar to how more facilitation strategies does not necessarily mean better implementation (because of the level of complexity), more “equitable” delivery does not necessarily mean better implementation. Understanding each population’s variability through exploration of high and low adopters for example, with in-depth interviews, can move towards an assessment of social determinants, and suggest recommendations for intervention adjustments that do not violate core components, but can address the context of the needs of each particular group. As well, studies of health coaching fidelity should include direct observation or audio/video recordings for assessment of the responsiveness of health coaches [34–36]. Completed calls and length of call are relatively weak measures of engagement or patient-centered counselling.

Technology can be a great enabler of care delivery, but if left unchecked, can also cause fidelity failure. To explore this topic we evaluated language as a moderator across a wide range of fidelity outcomes—for systems delivery and in-person health coaching touches. Based on these findings, we recommend consideration of language equity as a moderator in multi-lingual Health IT interventions, as it concerns whether an intervention is delivered equally across all populations (in our case between Spanish-speaking and English-speaking participants) over time. This work is unique in bringing together fidelity, health IT, equity, and health coaching but it also builds on existing implementation research to study how technology is implemented, to explore the impact on multi-lingual populations [37]. This study also highlights an approach to make more concrete existing fidelity frameworks with a step-wise approach outlined in the conceptual model. We hope this work can guide exploration of fidelity for health IT interventions, and in particular, those that include an automated ‘push’ along with a ‘personalized’ follow-up by a health coach or other health professional.

For low-income populations such as the women enrolled in this study, the underlying contributions of social determinants and structural barriers (such as limited economic resources, language barriers, or limited healthcare access), may impede engagement with health coaching programs if participants are not able to prioritize addressing their prevention-focused health needs in real time in addition to the other demands they face. It is critical to explore to what extent offering adapted multi-lingual interventions, especially those with health coaching components, are acceptable and to what extent modifiers may impact core fidelity measures. In regards to the value equation for this area of work [8], we believe that the value of providing this adapted version of the DPP, to include, for example infant care content, increased the acceptability of this program, particularly among Spanish speakers, who often are difficult to reach in post-partum care. For example, 100% of Spanish speakers reported the program worked well for infant care content, and 100% of Spanish-speakers reported they felt the program was respectful of their time. We believe that the participatory adaptation process undertaken with STAR MAMA was critical to successful engagement [38]. That we identified greater engagement and acceptability with the non-English speaking group is consistent with other work we and others have done regarding language and health coaching engagement [21, 39, 40].

## Conclusion

Implementation fidelity for health IT interventions should address moderating factors as well as systems level factors such as program delivery and ease of technology adoption. Application of modified fidelity frameworks that explicitly considers equity-based moderators can help ensure there is equitable delivery of interventions and promote the inclusion of a wider range of predictors to help understand variation in program uptake. Further work in the integration of equity into fidelity frameworks should consider how moderators such as language equity impact outcomes, for both specificed health outcomes and those associated with patient acceptability and program sustainability.

## Data Availability

The datasets that support the findings of this study are not publicly available due to information that could compromise research participant consent and privacy but can be made available from the corresponding author (MH) with appropriate precautions and upon reasonable request.

## Abbreviations

STAR MAMA: Support via Telephone Advice and Resources/Sistema Telefonico de Apoyo y Recoursos
Health IT: Health Information Technology
ATSM: Automated Telephonic Self-Management Support
